# Enhancement of Lutein Production in *Chlorella sorokiniana* (Chorophyta) by Improvement of Culture Conditions and Random Mutagenesis

**DOI:** 10.3390/md9091607

**Published:** 2011-09-20

**Authors:** Baldo F. Cordero, Irina Obraztsova, Inmaculada Couso, Rosa Leon, Maria Angeles Vargas, Herminia Rodriguez

**Affiliations:** 1 Institute of Plant Biochemistry and Photosynthesis, CIC Cartuja, University of Seville and CSIC, Avda. Americo Vespucio no. 49, 41092-Seville, Spain; E-Mails: baldomero@ibvf.csic.es (B.F.C.); irina@us.es (I.O.); inmaculada.couso@ibvf.csic.es (I.C.); avargas@us.es (M.A.V.); 2 Department of Chemistry, Experimental Sciences Faculty, University of Huelva, Avda. Fuerzas Armadas s/n, 21071-Huelva, Spain; E-Mail: rleon@uhu.es

**Keywords:** microalgae, carotenoids, batch culture, mixotrophic culture, *N*-methyl-*N*′-nitro-nitrosoguanidine, high lutein yielding mutants

## Abstract

*Chlorella sorokiniana* has been selected for lutein production, after a screening of thirteen species of microalgae, since it showed both a high content in this carotenoid and a high growth rate. The effects of several nutritional and environmental factors on cell growth and lutein accumulation have been studied. Maximal specific growth rate and lutein content were attained at 690 μmol photons m^−2^ s^−1^, 28 °C, 2 mM NaCl, 40 mM nitrate and under mixotrophic conditions. In general, optimal conditions for the growth of this strain also lead to maximal lutein productivity. High lutein yielding mutants of *C. sorokiniana* have been obtained by random mutagenesis, using *N*-methyl-*N*′-nitro-nitrosoguanidine (MNNG) as a mutagen and selecting mutants by their resistance to the inhibitors of the carotenogenic pathway nicotine and norflurazon. Among the mutants resistant to the herbicides, those exhibiting both high content in lutein and high growth rate were chosen. Several mutants exhibited higher contents in this carotenoid than the wild type, showing, in addition, either a similar or higher growth rate than the latter strain. The mutant MR-16 exhibited a 2.0-fold higher volumetric lutein content than that of the wild type, attaining values of 42.0 mg L^−1^ and mutants DMR-5 and DMR-8 attained a lutein cellular content of 7.0 mg g^−1^ dry weight. The high lutein yield exhibited by *C. sorokiniana* makes this microalga an excellent candidate for the production of this commercially interesting pigment.

## 1. Introduction

Carotenoids are synthesized by all photosynthetic organisms as well as by some non-photosynthetic bacteria and fungi. There are two main classes of naturally occurring carotenoids: carotenes, which are hydrocarbons, either linear or cyclic in either one or both ends of the molecule, and xanthophylls, which are oxygenated derivatives of carotenes. In microalgae, a distinction can be made between primary and secondary carotenoids. Primary carotenoids, as lutein, function as accessory pigments in the photosystems, as structural components of light harvesting complexes in chloroplasts, as well as photoprotective agents and therefore are essential for cell survival. Secondary carotenoids, such as astaxanthin, accumulate in large quantities in lipid bodies outside the chloroplasts, after subjecting cells to stress conditions. The role of secondary carotenoids in algal cells is not fully understood. They could function as photoprotective filters and as antioxidants preventing accumulation of oxygen radicals [1–3].

Lutein is used as a food dye and especially as a feed additive in aquaculture and poultry farming; it is also used for the coloration of pharmaceutical products and cosmetics [4]. Recently, additional applications for lutein, especially in the field of human health, have been found. Lutein is used as a nutraceutical against macular degeneration; lutein and zeaxanthin are known to play a critical function in maintaining a normal visual function [5–8]. In addition to the development of cataracts, also the progression of early atherosclerosis seems to be hampered by lutein [9,10]. In canines and cats, it has been proved that lutein enhances both cell mediated and humoral immune response [11,12]. Lutein has also been proposed for the prevention of certain cancers [13] and to protect skin from UV-induced damage [5]. Global lutein market has been increasing markedly in the last years [14]. In the US only, sales amount to $150 million [14]. Currently the commercial source of lutein is Marigold (*Tagetes erecta* and *Tagetes patula*) [14]. However, the lutein content of Marigold flowers is low (0.3 mg g^−1^ DW), and therefore there is an increasing interest in microalgae as an alternative source of this carotenoid [14,15]. The microalgae *Muriellopsis* sp. [16,17], *Chlorella zofingiensis* [18], *Coccomyxa acidophila* [19], *Scenedesmus almeriensis* [20] and *Chlorella protothecoides* [21] have been proposed as potential sources of lutein. Nevertheless, the described lutein values are not high enough to be economically feasible on an industrial scale. There is a need to improve lutein accumulation and productivity, by selecting an adequate species, optimizing culture conditions and obtaining high lutein yielding mutants.

Algal species with improved growth rate and enhanced carotenoid accumulation make the commercial process of lutein production more feasible. Induction and selection of mutants has been a technique widely employed for strain improvement as well as for studying the mechanisms of metabolic processes [22]. The introduction of a mutation in a certain carotenoid biosynthetic gene by isolating mutants resistant to a specific inhibitor for the carotenoid biosynthesis has been a method commonly used to obtain mutants of certain microalgae exhibiting high carotenoid contents. There are several reports describing the isolation of mutants of *Haematococcus pluvialis*, showing higher astaxanthin content per cell than the wild type by random mutagenesis with ethyl methane sulfonate (EMS) or UV and subsequent mutant selection on carotenoid biosynthesis inhibitors, such as compactin, nicotine, diphenylamine or norflurazon [23–25]. However, there is no available information on the obtention of high lutein yielding mutants most probably because cell growth is negatively affected in these mutants.

The present study describes: (1) The screening of thirteen different microalgae to select a good candidate for lutein production; (2) The effect of some nutritional and environmental factors on growth and lutein content in *Chlorella sorokiniana*, the selected species; and (3) The obtention of mutants of *C. sorokiniana* with high yields of lutein in comparison to the wild type strain by random chemical mutagenesis using MNNG.

## 2. Results

### 2.1. Screening of Different Species of Chlorophycean Microalgae for Lutein Production

Cell growth and carotenoid content of cultures of 13 chlorophycean microalgae are shown in Table 1. In all species lutein was the most abundant carotenoid. The highest lutein levels (24 mg L^−1^) were found in *C. sorokiniana*, *Monoraphidium braunii*, *Scenedesmus armatus* and *Scenedesmus vacuolatus* (Table 1). Other carotenoids like antheraxanthin, α-carotene, β-carotene, violaxanthin and zeaxanthin were also produced in all the tested species albeit at concentrations lesser than lutein. Astaxanthin and canthaxanthin were present only in *Chlorella fusca*, *Chlorella zofingiensis*, *Chlorococcum* sp. and *S. vacuolatus*. With regard to growth, *C. sorokiniana* and *S. armatus* exhibited the highest specific growth rates of 0.11 and 0.09 h^−1^, respectively, and for most species the maximum biomass value attained in the culture ranged between 7.5 to 8.5 g L^−1^, except for *Chlamydomonas reinhardtii*, *Chlorella luteoviridis* and *Chlorella stigmatofora*, which showed lower biomass values.

Some of the species considered in this work, such as *C. sorokiniana*, *M. braunii*, *S. armatus* and *S. vacuolatus* are of potential practical interest on the basis of their high lutein level. Among these, *C. sorokiniana* has been selected for further work focused on the production of lutein, since this microalga also shows the highest specific growth rate (0.11 h^−1^) and a high biomass value (8.0 g L^−1^).

### 2.2. Effect of Some Environmental and Nutritional Factors on Growth and Lutein Content in *C. sorokiniana*

#### 2.2.1. Growth and Lutein Accumulation under Standard Conditions

**Figure 1:**
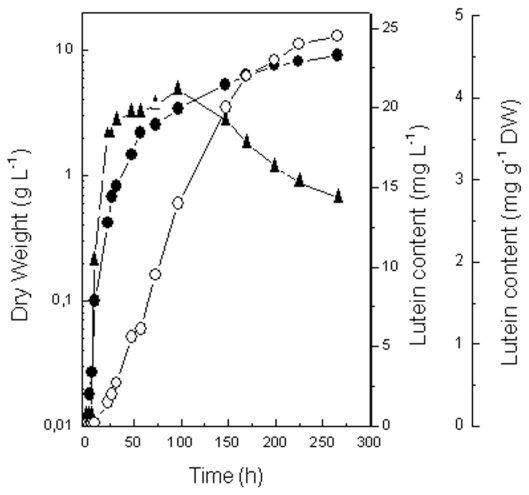
Kinetics of growth and lutein accumulation in a batch culture of *C. sorokiniana* under standard conditions. Culture conditions were the standard ones reported in Experimental Section. Data shown represent the mean values of three independent measurements, SD being lower than 10% of the means. Symbols: (closed circles) dry cell weight; (open circles) volumetric lutein content in the culture; (closed triangles) cellular lutein content.

Figure 1 shows the evolution with time of growth and lutein content in a photoautotrophic batch culture of *C. sorokiniana*. Both volumetric and cellular lutein content increased with cell biomass, attaining a maximum of 24 mg L^−1^ at the end of the deceleration phase, and 4.2 mg g^−1^ DW in the early deceleration phase, respectively. However, whereas the volumetric content was kept constant in the deceleration phase, the cellular content decreased markedly to 2.8 mg g^−1^ DW. The accumulation of the rest of carotenoids followed the same trend as lutein (data not shown).

#### 2.2.2. Effect of Irradiance

Cell growth and lutein accumulation in *C. sorokiniana* batch cultures at different irradiances, in the range from 92 to 1495 μmol photons m^−2^ s^−1^, was studied. Both specific growth rate and biomass in the culture increased with irradiance until 690 μmol photons m^−2^ s^−1^, by 2.6-fold and 1.5-fold, respectively, keeping constant at higher intensity values. Lutein content in the culture was enhanced by 87% as irradiance increased from 92 to 690 μmol photons m^−2^ s^−1^, decreasing at higher irradiance values. Cellular lutein content exhibited an optimum in an irradiance range between 368 and 690 μmol photons m^−2^ s^−1^, decreasing at higher and lower irradiances by 22 and 19%, respectively (Table 2). The maximum cellular lutein content (4.3 mg g^−1^ DW) was reached at 690 μmol photons m^−2^ s^−1^, in the early deceleration phase of growth (data not shown).

#### 2.2.3. Effect of Temperature

The influence of temperature on lutein level and growth of *C. sorokiniana* has also been examined. Cell growth, in terms of specific growth rate, increased by 50% when the temperature was raised from 25 to 28 °C, keeping constant at higher temperatures. Biomass in the culture increased by about 10% with temperatures up to 28 °C, decreasing by 44% at higher temperatures. Optimal volumetric and cellular lutein contents were recorded in a range from 28 to 32 °C, decreased values being registered at lower and higher temperatures (Table 3). The maximum cellular lutein content (4.2 mg g^−1^ DW) was achieved at 28 °C in the early deceleration phase of growth (data not shown).

#### 2.2.4. Effect of Nitrogen

Nitrogen availability affects carotenoid accumulation in some microalgae [14], therefore the effect of nitrate concentration on lutein production was also assayed. Both specific growth rate and biomass in the culture increased by 30% when nitrate concentration in the medium was enhanced from 10 to 40 mM, decreasing by about 15% at higher concentrations. In addition, volumetric and cellular lutein contents increased 2.9 and 2.3-fold, respectively, when nitrate concentration in the medium was raised from 10 to 40 mM, decreasing by about 10% at higher nitrate concentrations (Table 4). Nevertheless, maximum cellular lutein content did not change significantly with nitrogen concentration in the range from 20 to 80 mM (data not shown).

#### 2.2.5. Effect of NaCl

The influence of NaCl concentration in the medium was also analyzed in a range from 2 to 200 mM. Both growth and lutein content decreased drastically at NaCl concentrations higher than 2 mM (data not shown).

#### 2.2.6. Effect of Acetate and Glucose (Mixotrophic Culture)

The main limiting factor for biomass productivity in photoautotrophic cultures of microalgae is imposed by light availability. To solve this problem mixotrophic cultures can be used. For that reason the effect of acetate, as an extra source of carbon, on growth and lutein accumulation was assayed in *C. sorokiniana*. As shown in Table 5, specific growth rate and biomass in the culture were enhanced by 33% and 20%, respectively, when acetate concentration in the medium was raised from 0 to either 40 or 50 mM, decreasing only by 8% thereafter. The volumetric and cellular lutein contents increased by 45% and 20%, respectively, when acetate was added to the medium at concentrations up to 40 mM, decreasing by a 25% at higher acetate concentrations. The maximum cellular lutein content followed the same trend as cellular lutein content at the end of the deceleration phase, attaining maximum values of 5.2 mg g^−1^ DW at 40 mM of acetate (data not shown).

The addition of glucose, as the only extra carbon source, to cultures of *C. sorokiniana* increased by 60% the biomass in the culture and by 12% the volumetric lutein content, which attained a level of 33.5 mg L^−1^, the cellular lutein content decreasing by 30% (Table 6). A similar effect was observed when glucose was added to cultures containing 40 mM acetate before the end of the exponential phase, reaching a volumetric lutein content of 35.0 mg L^−1^. On the other hand, an extra addition of acetate or sodium nitrate before the end of the exponential phase did not affect either biomass or lutein content significantly. Moreover, when sodium nitrate was replaced by urea, the specific growth rate and lutein content were decreased by 17% and 20%, respectively.

Similar values of growth and lutein content were registered when sodium nitrate was replaced by ammonium nitrate. When *C. sorokiniana* was grown heterotrophically in the dark with glucose as the only carbon source, specific growth rate was similar and biomass decreased only slightly with regard to the values obtained under mixotrophic conditions, however lutein accumulation was much lower than that measured in mixotrophic or photoautotrophic conditions (data not shown).

### 2.3. Isolation of High Lutein Yielding Mutants of *C. sorokiniana*

**Figure 2:**
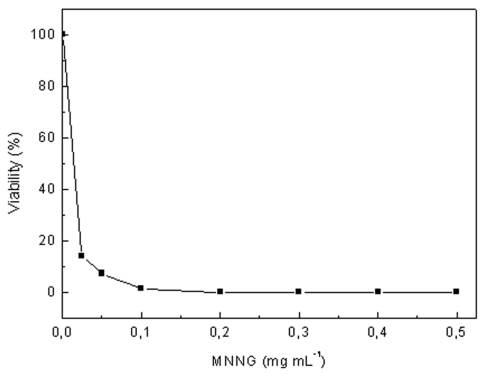
Survival curve of *C. sorokiniana* to the chemical mutagen MNNG. Data shown represents mean values of three independent measurements, SD being lower than 10%.

To obtain high lutein producing mutants of *C. sorokiniana*, cells were subjected to random chemical mutagenesis with MNNG and mutants were screened on the basis of their resistance either norflurazon or nicotine, growth rate and lutein content. First, the survival curve for mutagenesis with MNNG was performed to determine the MNNG concentration which resulted in around 5–10% of cells viability (Figure 2), and afterwards, a wide range of concentrations of the herbicides were tested to find out the minimal concentration which inhibited the grown of the wild strain, resulting in 400 μM for nicotine and 4 μM for norflurazon (data not shown).

According to the first step of selection, 745 herbicide-resistant colonies were obtained, from which only 222 (210 resistant to norflurazon and 12 resistant to nicotine) were selected according to the second step criterion, which consisted of discarding mutants exhibiting either low growth or unstable resistance to the herbicides. The selected mutants were grown in shaken liquid cultures under photoautotrophic standard conditions to determine lutein content and growth rate. Increases in volumetric and cellular lutein contents of the best mutants as compared to the wild type are shown in Table 7. The mutant MR-16, resistant to nicotine, exhibited volumetric and cellular lutein contents 2.0- and 1.4-fold higher, respectively, than those of the wild strain. This mutant also showed a 3.0-fold higher volumetric α-carotene and antheraxanthin levels and 2.0-fold higher β-carotene and zeaxanthin contents as compared with those of the wild type, being the total volumetric carotenoid content 2.0-fold higher than in the wild strain (data not shown). The mutants DMR-5 and DMR-8, both resistant to norflurazon, stood out in terms of cellular content in lutein, showing 53–55% increase relative to the wild type, reaching values of 7.0 mg lutein g^−1^ DW.

**Figure 3:**
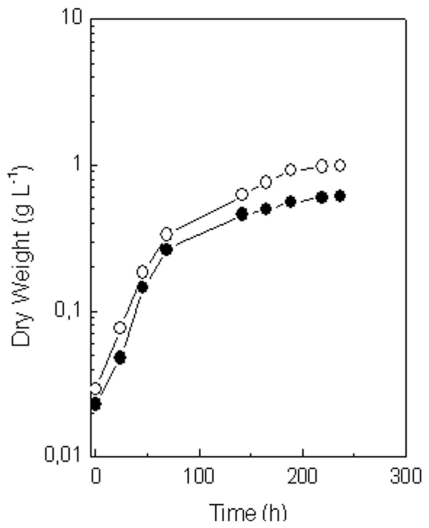
Kinetics of growth of *C. sorokiniana* wild type and the mutant MR-16. Cells were grown as indicated in Table 7. Data shown represent mean values of three independent measurements, SD being lower than 10%. Symbols: (closed circle) wild type; (open circles) mutant MR-16.

Moreover, the growth of the selected high lutein producing mutants under photoautotrophic conditions was similar or even higher than that of the parental strain, as shown in the Figure 3 for MR-16.

All mutants of *C. sorokiniana* obtained in the processes of mutagenesis were analyzed for their viability and stability by consecutive sub-cultures in nonselective and selective media with herbicides to check their resistance.

### 2.4. Comparison of Growth and Lutein Accumulation in the Selected Mutant (MR-16) and in the Wild Type of *C. sorokiniana* under Best Photoautotrophic and Mixotrophic Culture Conditions

MR-16 mutant exhibited both a growth and lutein content higher than the wild strain under photoautotrophic conditions (Figure 3, Table 7). For this reason, cells of the wild type and the MR-16 mutant were grown under the best photoautotrophic and mixotrophic conditions found previously in the wild strain of *C. sorokiniana* for lutein accumulation in order to know accurately the improvement of the MR-16 mutant as compared to the wild type. As shown in Table 8, the volumetric and cellular lutein contents increased both by 68% in the mutant MR-16 with respect to the wild strain when the mutant was grown photoautotrophically, attaining maximum values of 42.0 mg L^−1^ and 5.0 mg g^−1^ DW at the end of the deceleration phase. On the other hand, specific growth rate was either constant or very similar at the different conditions assayed in both strains. However when the mutant was grown mixotrophically, biomass decreased 40% as compared to the wild strain. The addition of acetate and glucose to cells of the MR-16 mutant had a negative effect decreasing a 48% the maximum lutein content in the culture, and a 33% the cellular content with regard to the parental strain. A difference between the wild and the mutant *C. sorokiniana* strains was that the wild type showed the maximum values of cellular lutein during the early deceleration phase, whereas MR-16 mutant attained these contents at the end of the deceleration phase, due to a continuous and higher lutein accumulation.

## 3. Discussion

### 3.1. Effect of Several Environmental and Nutritional Factors on Lutein Production by *C. sorokiniana* under Photoautotrophic Conditions

Since irradiance, temperature, NaCl and nitrate concentration are known to affect the levels of different carotenoids in microalgae, the effect of these factors on cell growth and lutein accumulation was analyzed in *C. sorokiniana* which has been selected among thirteen chlorophycean microalgae, since it showed the highest growth rate and lutein accumulation. In this selected microalga lutein content in the culture was optimal at moderate irradiances, since both biomass and cellular content in lutein decreased at both high and low irradiance (Table 2). A similar trend with regard to the cellular content has also been observed for lutein and β-carotene in different species of *C. zofingiensis* and *Muriellopsis* sp. [26,16,18]. In contrast, in *H. pluvialis* and *C. zofingiensis* the cellular content of secondary carotenoids, such as astaxanthin and canthaxanthin, followed the opposite trend to lutein, being enhanced at high irradiance [18,27]. *C. sorokiniana* exhibited maximal volumetric and cellular lutein content in the range of 28 to 32 °C decreasing at lower and higher temperatures (Table 3); these results being in agreement with those obtained in the lutein-producing microalga *C. zofingiensis* [16] and *Scenedesmus almeriensis* [20,28]. Contrastingly, extreme temperatures triggered carotenogenesis in *H. pluvialis*. It has been suggested that endogenously generated active oxygen is responsible for the stimulation of astaxanthin synthesis at high temperature in this microalga [24]. Nitrogen limitation enhances the synthesis of secondary carotenoids such as astaxanthin, but reduces biomass yield. However, nitrogen at none limiting concentrations is required for primary carotenoids accumulation due possibly to the need of a continued synthesis of light-harvesting proteins and structural xanthophylls under optimal growth conditions [3,16,18]. This agrees with the results obtained in *C. sorokiniana* since both volumetric and cellular lutein content increased when nitrate concentration in the culture medium was raised from 10 to 40 mM, decreasing slightly at higher nitrate concentrations (Table 4). On the other hand, although in heterotrophic cultures of *C. protothecoides* maximal lutein productivities were achieved using urea as the nitrogen source [29], in *C. sorokiniana* grown mixotrophically either a slight decrease or no effect in growth and lutein content was registered when sodium nitrate was replaced by either urea (Table 6) or ammonium nitrate (data not shown). NaCl stress, which has been described to induce the biosynthesis of secondary carotenoids [30], seems not to trigger the biosynthesis of the primary ones. Thus in *C. sorokiniana*, both growth and lutein content decreased drastically when NaCl concentrations were increased (data not shown), and in the cases of other chlorophyta as *Muriellopsis* sp., *C. zofingiensis* and *S. almeriensis*, the lutein levels per cell remained practically constant at the different NaCl concentrations assayed [16,18,20]. Therefore, although stress factors, such as high irradiance, extreme temperatures, high NaCl concentration or nutrients limitation enhance the cellular accumulation of secondary carotenoids, such as astaxanthin [1,18,31], these factors do not increase the cellular levels of lutein in *C. sorokiniana*, since lutein is a primary carotenoid, being required for the structure and function of the light-harvesting complexes in photosynthesis [2], and accordingly, conditions that increase photoautotrophic growth of this microalga are also those enhancing lutein accumulation.

### 3.2. Lutein Production by *C. sorokiniana* under Mixotrophic Conditions

In photoautotrophic mass cultures of microalgae for the production of biomass and valuable compounds, the main limiting factor is usually light availability, which in many cases limits cell density and productivity of the cultures, making it unprofitable for industry. An alternative to overcome this problem is to use mixotrophic cultures. In *C. sorokiniana*, the addition of acetate to the cultures enhanced both growth and volumetric and cellular lutein contents (Table 5). Although it is known that acetate enhances growth and synthesis of astaxanthin in *H. pluvialis* [32], the effect of this carbon source on the biosynthesis of lutein has not been studied in other microalgae. Even though the addition of glucose decreased the cellular content in lutein, it increased biomass considerably; therefore, the volumetric lutein content was enhanced and the highest volumetric lutein levels (35 mg L^−1^) were achieved in cultures supplemented with this carbon source (Table 6). Therefore the supply of an extra carbon source to cultures supported higher growth and productivity, overcoming the limitation by light. Recently, it has been shown that in heterotrophically grown *C. zofingiensis*, glucose increased the cellular accumulation of astaxanthin and zeaxanthin by increasing the transcription levels of both β-carotene ketolase and β-carotene hydroxylase involved in the synthesis of these carotenoids, but decreased the cellular content of lutein and chlorophyll [33,34] and in *C. protothecoides* lutein productivity was much higher in heterotrophic cultures supplemented with glucose than in photoautotrophic cultures, since in the former very high cell densities were achieved [35]. Therefore, our results are in agreement with these findings, which indicate that although glucose decreases the celular content of lutein, it increases considerably the biomass, therefore resulting in a higher lutein production in mixotrophic cultures as compared to those performed photoautotrophically.

### 3.3. Enhancement of Lutein Yield in *C. sorokiniana* by Random Mutagenesis

There are few reports concerning the isolation of high carotenoid yielding mutants of microalgae by random mutagenesis. A mutant of *Chlorella regularis* showing a high cellular content of lutein has been isolated by Ishikawa *et al.* (2004) [36], although it exhibited lower growth than the wild strain and no volumetric lutein content data have been reported. Some mutants with enhanced accumulation of astaxanthin have also been described, however their growth rates are usually lower than that of the wild type [37,23,38]. In our experiments with *C. sorokiniana*, mutants were selected not only on the basis of high lutein content, but also according to a high growth rate. Thus, the mutant MR-16 exhibited a growth and cellular lutein content higher than those of the wild type and, as a consequence, lutein yields 2.0-fold higher than those measured in the wild strain were achieved (Table 7 and Figure 3). In addition, this mutant shows the highest lutein content described in the literature under photoautotrophic conditions of growth at laboratory scale [14,16,18–20], attaining a value of 42.0 mg L^−1^ at the end of the deceleration phase (Table 8). The mutant MR-16 was resistant to nicotine, a specific inhibitor for the enzyme lycopene β-cyclase, involved in lutein biosynthesis, which could possibly have an altered specific activity in this mutant, for instance a modified structure for the herbicide-binding site, and/or a different expression of this enzyme, which means a higher enzyme activity, and would result into an improved lutein production under photoautotrophic growth. In addition, the higher growth rate of this mutant with respect to the wild type strain under photoautotrophic conditions could be due to the higher cellular lutein content, since this carotenoid plays important roles in the function, structure and photo-protection of the photosynthetic apparatus, leading to a higher photosynthetic efficiency. It is relevant to mention that the addition of acetate and/or glucose to the culture medium decreased the biomass in the cultures of MR-16. The mechanism of this effect is not understood yet. It is possible that the mutation has provoked a metabolic alteration causing a weak repression of growth by glucose or acetate.

### 3.4. *C. sorokiniana* as a Promising Lutein Producer for Commercial Applications

Although microalgae are not used yet as a lutein source at industrial scale, first steps have been made at laboratory and pilot scales in the recent years [16,14,28,21]. The data here reported point to *C. sorokiniana* as an attractive candidate for the production of lutein, since it shows a high growth rate (0.12 h^−1^), a volumetric lutein content of 35 mg L^−1^ and cellular lutein contents of 5.2 mg g^−1^ DW, which are enhanced by random mutagenesis up to 42.0 mg L^−1^ and 7.0 mg g^−1^ DW. These values are similar or higher than those reported for other lutein producing microalgae grown at laboratory scale under photoautotrophic conditions. Thus, *C. zofingiensis* shows a specific growth rate of 0.04 h^−1^ and a volumetric and cellular lutein content of 20.0 mg L^−1^ and 3.0 mg g^−1^ DW, respectively [18,14]; *Scenedesmus almeriensis* has been reported to accumulate 5.5 mg g^−1^ DW and a specific growth rate of 0.07 h^−1^ [20]; *Muriellopsis* exhibited a growth rate of 0.17 h^−1^ and a volumetric and cellular lutein content of 29.0 mg L^−1^ and 5.5 mg g^−1^ DW, respectively [16]; and *Coccomyxa acidophila* has been reported to accumulate 6.1 mg g^−1^ DW under extreme culture conditions [19]. From all this information, we can propose C*. sorokiniana* as a promising microalgal species for the production of lutein for commercial applications.

## 4. Experimental Section

### 4.1. Organisms

The species of microalgae used in this work were: *Chlorella fusca* 211-8b, *Chlorella sorokiniana* 211-32, *Chlorella zofingiensis* 211-14, *Monoraphidium braunii* 202-7d, *Scenedesmus vacuolatus* 211-15 and *Chlorella stigmatofora* 9–86 from SAG, Culture Collection of Göttingen University (Germany); *Chlorella luteoviridis* 258, *Chlorococcum* sp. 2438, *Scenedesmus armatus* 2533, *Scenedesmus quadricauda* 76 and *Scenedesmus obliquus* 393 from UTEX, Culture Collection of Algae of the University of Texas (USA); *Chlorella vulgaris* 101 from UAM, Culture Collection of Microalgae of Universidad Autónoma de Madrid (Spain); and *Chlamydomonas reinhardtii* CC621(−) from Culture Collection of Institut für Biologie III at the University of Freigburg (Germany).

### 4.2. Culture Conditions

#### 4.2.1. Standard Culture Conditions

Cells were grown photoautotrophically by bubbling through the cell suspension air supplemented with 1% (v/v) CO_2_ as the only source of carbon. The culture medium of Arnon *et al.* (1974) [39] modified to contain 4 mM K_2_HPO_4_ and 20 mM NaNO_3_, was used. The cells were grown in batch culture at 28 °C, in Roux flasks of 1 L capacity, laterally illuminated with mercury halide lamps at 460 μmol photons m^−2^ s^−1^, measured at the surfaces of the flasks using a LI-COR quantum sensor (model L1-1905B, Li-Cor, Inc. Lincoln, NE, USA) connected to a quantum photometer.

#### 4.2.2. Mixotrophic Culture Conditions

Cells were grown mixotrophically by the addition to Arnon medium (modified to contain 4 mM K_2_HPO_4_ and 20 mM NaNO_3_) sodium acetate and/or glucose, either at the beginning of the culture or after 24 h (by the middle of the exponential phase) and at an irradiance of either 460 or 690 μmol photons m^−2^ s^−1^. The rest of culture conditions were the same as the standard ones.

### 4.3. Random Mutagenesis and Selection of High Lutein Yielding Mutants of *C. sorokiniana*

Cells of *C. sorokiniana* in the exponential phase of growth (10^6^ cells mL^−1^) were harvested by centrifugation (2700× *g*, 10 min), washed with sterile water and treated with 0.1 mg mL^−1^ of 1-methyl-3-nitro-1-nitrosoguanidine (MNNG) (survival rate 5–10%) for 1 h. This mutagen is known to induce nucleotides substitutions at high frequencies and little lethality, and the inhibitors nicotine and norflurazon were used as the selection method. These herbicides inhibit the carotenogenic enzymes lycopene β-cyclase and phytoene desaturase, respectively. Therefore, the screening of the mutants was performed by their resistance to the carotenoid biosynthesis inhibitors nicotine and norflurazon.

The treated cells were washed with sterile water, resuspended in Arnon modified medium and incubated under dim light during 24 h. After the incubation, cells were spread on solid modified Arnon medium containing, either 4 μM norflurazon (Supelco, Bellefonte, PA, USA) or 400 μM nicotine (Sigma-Aldrich, Steinheim, Germany) and incubated at 25 °C and 50 μmol photons m^−2^ s^−1^ for 3–4 weeks. Then, the herbicide-resistant colonies were sub-cultivated several times in solid medium containing either norflurazon or nicotine to check their resistance to the herbicide and growth in solid medium. Herbicide-resistant mutants that showed good growth were grown in 100 mL-capacity erlenmeyers under photoautotrophic conditions, shaken at 100 rpm and illuminated from the top at 50 μmol photons m^−2^ s^−1^, in order to analyze carotenoids content and growth.

### 4.4. Analytical Methods

For dry weight (DW) determinations, 5 mL aliquots of the cell culture were filtered through pre-dried Whatman GF/C paper (Whatman International Ltd., Maidstone, England), washed three times with distilled water, and the filters containing the algae were dried at 80 °C for 24 h.

Specific growth rate (μ) was calculated from the measured DW during the exponential phase of growth, using the equation: μ = (ln *x*_2_ − ln *x*_1_)/(*t*_2_ − *t*_1_), where *x*_2_ and *x*_1_ represent DW values in terms of g L^−1^ at times *t*_2_ and *t*_1_, respectively.

For carotenoid analysis, pigments were extracted with methanol at 70 °C, centrifuged, the supernatant evaporated under N_2_ and the pellet dissolved in methanol. Then the samples were centrifuged and analyzed by HPLC using a Waters Spherisorb ODS2 column (4.6 × 250 mm, 5 μm particle size) (Waters, Mildford, MA, USA). The chromatographic method described by Cordero *et al.* [40] was used. The pigments were eluted at a flow rate of 1.2 mL min^−1^ and detected at 440 nm using a Waters 2996 photodiode-array detector. Identification of carotenoids was achieved by comparison of the individual characteristic absorption spectrum and the retention time with known standards. Quantification was performed using a calibration curve generated with commercially available carotenoids standards from Sigma-Aldrich (St. Louis, MO, USA) and DHI (Holsholm, Germany).

Samples analyzed were withdrawn along the curve of growth. Independent triplicate analyses were carried out for each sample, the results representing the mean values. The standard deviation (SD) is omitted since it was lower than 10% of the mean values.

## 5. Conclusions

*Chlorella sorokiniana* has been selected for lutein production after a screening of thirteen species of microalgae. The effects of several nutritional and environmental factors on cell growth and lutein content have been studied. Maximal specific growth rate and lutein accumulation were attained at 690 μmol photons m^−2^ s^−1^, 28 °C, 2 mM NaCl, 40 mM nitrate and under mixotrophic conditions, attaining values of lutein of 35.0 mg L^−1^ and 5.2 mg g^−1^ DW. In general, optimal conditions for the growth of this species also lead to maximal lutein productivity, since lutein is a primary carotenoid, being required for the structure and function of the light-harvesting complexes in photosynthesis. These lutein values were further enhanced by chemical random mutagenesis up to 42.0 mg L^−1^ and 7.0 mg g^−1^ DW, using MNNG and selecting mutants by: (1) their resistance to the inhibitors of the carotenogenic pathway nicotine and norflurazon; (2) their high growth rate; and (3) high lutein content. From all our results we can propose *C. sorokiniana* as an interesting and promising microalga for the production of lutein for commercial and industrial applications.

## Figures and Tables

**Table 1:** Specific growth rate (μ), biomass and carotenoids levels in cultures of several species of chlorophycean microalgae. Culture conditions for all the species were the standard ones as reported in the Experimental Section. Data correspond to the maximal values attained in the culture at the deceleration phase: 9–10 days (^a^), 11–12 days (^b^) and 13–14 days (^c^) of culture, being the means of three independent measurements. The standard deviation (SD) is omitted since it was lower than 10% of the mean values. Carotenoids: Ant, antheraxanthin; Ast, astaxanthin; C, canthaxanthin; α-c, α-carotene; β-c, β-carotene; L, lutein; V, violaxanthin; Z, zeaxanthin. nd: not detected.

Species	μ (h^−1^)	Biomass (g L^−1^)	Carotenoid Content (mg L^−1^)							
			Ant	Ast	C	α-c	β-c	L	V	Z
*Chlamydomonas reinhardtii*^b^	0.05	4.5	0.4	nd	nd	0.1	3.0	12.4	1.0	0.2
*Chlorella fusca*^b^	0.04	8.5	1.5	6.5	1.2	0.3	4.6	22.0	1.5	6.0
*Chlorella luteoviridis*^b^	0.03	2.7	0.3	nd	nd	nd	0.5	5.8	0.2	0.6
*Chlorella sorokiniana*^a^	0.11	8.0	0.3	nd	nd	0.2	1.6	24.0	0.9	0.4
*Chlorella stigmatofora*^b^	0.02	4.1	0.1	nd	nd	0.1	nd	3.2	0.2	0.4
*Chlorella vulgaris*^c^	0.08	8.5	0.9	nd	nd	1.8	2.3	22.2	2.2	3.3
*Chlorella zofingiensis*^c^	0.03	7.6	0.9	5.6	1.2	0.4	3.0	20.0	1.3	3.0
*Chlorococcum* sp. ^a^	0.03	7.5	0.8	nd	0.8	0.2	2.2	15.0	2.5	0.5
*Monoraphidium braunii*^c^	0.07	8.4	2.4	nd	nd	0.9	3.8	24.0	2.1	8.4
*Scenedesmus armatus*^b^	0.09	8.5	0.5	nd	nd	0.3	4.4	24.0	2.4	nd
*Scenedesmus quadricauda*^c^	0.06	8.4	0.6	nd	nd	0.3	3.0	22.0	5.0	nd
*Scenedesmus obliquus*^b^	0.08	8.0	0.5	nd	nd	0.2	2.5	15.0	1.5	1.2
*Scenedesmus vacuolatus*^c^	0.08	8.2	1.6	5.3	1.7	0.5	4.2	24.0	2.4	3.8

**Table 2:** Effect of irradiance on growth and lutein accumulation in *C. sorokiniana*. Cells were grown at the indicated irradiances, the rest of culture conditions being the standard ones described in Experimental Section. Biomass and lutein content data correspond to the mean values of three independent measurements recorded after 10 days, at the end of the deceleration phase, when the maximal volumetric lutein contents and biomass values were attained in the cultures. The SD were lower than 10% of the means.

Irradiance (μmol photons m^−2^ s^−1^)	μ (h^−1^)	Biomass (g L^−1^)	Lutein Content	
			mg L^−1^	mg g^−1^ DW
92	0.05	6.0	15.0	2.5
230	0.08	7.2	19.0	2.6
368	0.11	7.8	23.0	3.0
460	0.11	8.0	24.0	3.0
690	0.13	9.0	28.0	3.1
920	0.13	8.5	24.0	2.8
1495	0.13	8.5	20.0	2.4

**Table 3:** Effect of temperature on growth and lutein accumulation in *C. sorokiniana*. Cells were grown at the indicated temperatures, the rest of culture conditions being the standard ones described in Experimental Section. Biomass and lutein content data are the mean values of three independent measurements recorded after 9 days, when cultures were at the end of deceleration phase, which corresponds to the maximal volumetric lutein contents and biomass attained in the cultures. The SD were lower than 10% of the means.

Temperature (°C)	μ (h^−1^)	Biomass (g L^−1^)	Lutein Content	
			mg L^−1^	mg g^−1^ DW
22	0.08	7.5	19.0	2.4
25	0.08	7.9	20.0	2.4
28	0.12	8.2	25.0	3.0
32	0.12	6.6	24.0	3.6
36	0.11	5.6	19.0	3.4
40	0.11	4.6	16.0	3.4

**Table 4:** Effect of nitrate concentration on growth and lutein accumulation in *C. sorokiniana*. Cells were grown at the indicated concentrations of NaNO_3_, the rest of culture conditions being the standard ones described in Experimental Section. Biomass and lutein content data are the mean values of three independent measurements recorded after 10 days, when cultures were at the end of the deceleration phase, which corresponds to the maximal volumetric lutein contents and biomass attained in the cultures. The SD were lower than 10% of the means.

NaNO_3_ concentration (mM)	μ (h^−1^)	Biomass (g L^−1^)	Lutein Content	
			mg L^−1^	mg g^−1^ DW
10	0.10	6.5	9.0	1.4
20	0.10	8.0	24.0	3.0
30	0.12	8.3	25.0	3.0
40	0.13	8.2	26.0	3.2
60	0.12	7.9	25.0	3.2
80	0.12	7.4	24.0	3.2
100	0.11	7.7	24.0	3.1
120	0.11	7.2	22.0	3.0

**Table 5:** Effect of acetate concentration on growth and lutein accumulation in *C. sorokiniana*. Cells were grown mixotrophically by the addition of sodium acetate at the indicated concentrations at the beginning of the culture, the rest of culture conditions being as described in Experimental Section. Biomass and lutein content data are the mean values of three independent measurements recorded after 10 days, when cultures were at the end of deceleration phase, which corresponds to the maximal volumetric lutein contents and biomass attained in the cultures. The SD were lower than 10% of the means.

Acetate concentration (mM)	μ (h^−1^)	Biomass (g L^−1^)	Lutein Content	
			mg L^−1^	mg g^−1^ DW
0	0.09	7.5	22.0	3.0
20	0.11	7.6	26.0	3.4
30	0.11	8.0	28.0	3.5
40	0.12	9.0	32.0	3.6
50	0.12	9.0	26.0	2.9
60	0.11	9.2	25.0	2.7

**Table 6:** Effect of carbon or nitrogen extra supply on growth and lutein accumulation in *C. sorokiniana*. Cells were grown mixotrophically at an irradiance of 690 μmol photons m^−2^ s^−1^ in Arnon medium modified to contain 4 mM K_2_HPO_4_, 40 mM sodium nitrate and 40 mM sodium acetate (AM), except where indicated (^b,c^).

Culture medium	μ (h^−1^)	Biomass (g L^−1^)	Lutein Content	
			mg L^−1^	mg g^−1^ DW
AM	0.12	8.8	30.0	3.4
AM + 100 mM Glucose a	0.11	13.5	35.0	2.6
AM + 40 mM Acetate a	0.11	9.0	29.0	3.2
AM + 40 mM Nitrate a	0.12	8.8	29.0	3.3
AM + 60 mM Urea b	0.10	8.5	23.0	2.7
AM + 100 mM Glucose c	0.11	14.0	33.5	2.4

a Addition of glucose, extra nitrate or acetate during exponential phase after 24 h of culture.

b Nitrate was replaced by urea at the beginning of the culture.

c Acetate was replaced by glucose at the beginning of the culture.  The rest of culture conditions were as described in Experimental Section. Biomass and lutein content data are the mean values of three independent measurements recorded after 10 days, when cultures were at the end of the deceleration phase, which corresponds to maximal volumetric lutein contents and biomass attained in the cultures. The SD were lower than 10% of the means.

**Table 7:** Increase in lutein content of the best mutants of *C. sorokiniana* relative to the wild type. Cells were grown photoautotrophically in shaken cultures at 50 μmol photons m^−2^ s^−1^, the rest of conditions being the standard ones described in Experimental Section. Data are mean values of three independent measurements in the deceleration phase after 13 days of growth, SD being lower than 10%. NF: resistant to norflurazon; NIC: resistant to nicotine.

Strain	Volumetric Lutein Content (mg L^−1^)	Cellular Lutein Content (mg g^−1^ DW)

	(% with respect to the wild strain)	
MR-3 (NF)	52	17
MR-14 (NIC)	63	29
MR-16 (NIC)	101	42
DMR-4 (NIC)	62	38
DMR-11 (NIC)	49	49
DMR-5 (NF)	29	55
DMR-8 (NF)	29	53

**Table 8:** Growth and lutein accumulation in MR-16 mutant and wild strain of *C. sorokiniana* under photoautotrophic and mixotrophic conditions. Cells were grown photoautotrophically at an irradiance of 690 μmol photons m^−2^ s^−1^ in Arnon medium modified to contain 4 mM K_2_HPO_4_ and 40 mM sodium nitrate, and mixotrophycally by the addition of both 40 mM sodium acetate and 100 mM glucose after 24 h of culture (by the middle of the exponential phase). The rest of culture conditions were as described in Experimental Section. Biomass and lutein content data are the mean values of three independent measurements recorded after 10 days, when cultures were at the end of the deceleration phase, which corresponds to maximal volumetric lutein content and biomass attained in the cultures. The SD were lower than 10% of the means.

Culture condition	μ (h^−1^)	Biomass (g L^−1^)	Lutein Content	
			mg L^−1^	mg g^−1^ DW
***Wild type***				
Photoautotrophic	0.11	8.4	25.0	3.0
Mixotrophic	0.11	13.0	33.0	2.6
***MR-16 mutant***				
Photoautotrophic	0.12	8.3	42.0	5.0
Mixotrophic	0.11	7.8	22.0	2.8
